# Supplementary material for: The Construction and Exploration of a Comprehensive MicroRNA Centered Regulatory Network in Foxtail Millet (Setaria italica L.)
Source: Front Plant Sci. 2022 May 6;13:848474. doi: 10.3389/fpls.2022.848474 (PMC9121102; doi:10.3389/fpls.2022.848474)
Supplement: Supplementary file 4 [file Data_Sheet_4.zip › Sit-miRNovel5_Seita.2G297300.1_1964_TPlot.pdf]

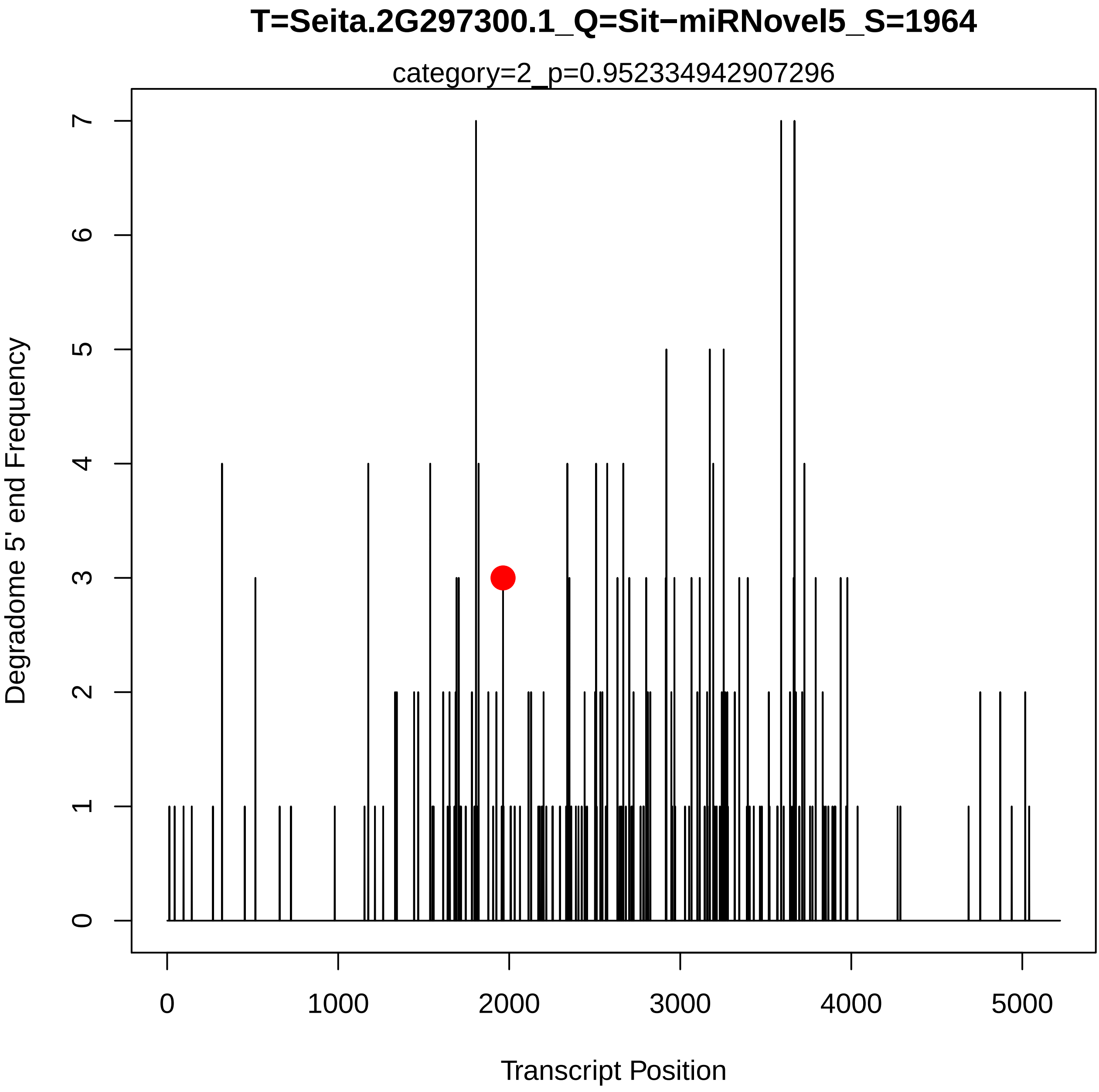
T=Seita.2G297300.1_Q=Sit−miRNovel5_S=1964
category=2_p=0.952334942907296
Degradome 5' end Frequency
Transcript Position
0
1
2
3
4
5
6
7
0
1000
2000
3000
4000
5000
